# Determination of specific life changes on psychological distress during the COVID-19 pandemic

**DOI:** 10.1371/journal.pone.0256481

**Published:** 2021-08-30

**Authors:** Keiko Kabasawa, Junta Tanaka, Tomoyo Komata, Katsuhiro Matsui, Kazutoshi Nakamura, Yumi Ito, Ichiei Narita

**Affiliations:** 1 Department of Health Promotion Medicine, Niigata University Graduate School of Medical and Dental Sciences, Niigata, Japan; 2 Department of Humanities, Faculty of Humanities, Niigata University, Niigata, Japan; 3 Division of Preventive Medicine, Niigata University Graduate School of Medical and Dental Sciences, Niigata, Japan; 4 Division of Clinical Nephrology and Rheumatology, Niigata University Graduate School of Medical and Dental Sciences, Niigata, Japan; National Cheng Kung University College of Medicine, TAIWAN

## Abstract

The COVID-19 pandemic might affect many aspects of the community and a range of psychiatric risk factors due to life changes, including people’s behaviors and perceptions. In this study, we aim to identify specific life changes that correlate with psychological distress within the social context of the COVID-19 pandemic in Japan. In July 2020, workers (company employees and civil servants) in Japan were recruited from local institutions that had not had any confirmed COVID-19 cases as well as neighborhoods that had only a few cases. Participants completed a COVID-19 mental health survey (*N* = 609; 66.9% male). Psychological distress was identified based on Kessler-6 scores (≥13). Life changes were assessed by an open-ended question about life changes in participants and their family, workplace, and community due to the COVID-19 pandemic. A convergent mixed-method approach was used to compare the context of perceived life changes in participants with psychological distress and those without. As a result, 8.9% of participants had psychological distress, and sex and age categories were different between those with psychological distress and those without. Among the participants who responded to the open-ended question, the biggest life change was “staying at home,” and the next biggest life changes were “event cancellations” and “increased workload” in participants with psychological distress, and “no changes” and “mask-wearing” in those without psychological distress, respectively. Regarding emotional/perceptual changes, “stress,” “fear,” and “anger” were more frequently reported by participants with psychological distress than those without (*P* <0.001). By integrating these findings, we identified themes focusing on vulnerable characteristics related to psychological distress. This study may provide a source in society for mediating psychological distress during a pandemic.

## Introduction

A pandemic prompts public health measures for individuals and changes in societal behaviors to prevent microbiome transmission [1]. Although people realize the importance of hygiene and health behaviors, society is nevertheless noted by fear and confusion. Indeed, coronavirus disease 2019 (COVID-19) rapidly spread worldwide [2], causing changes in people’s lives and behaviors, such as quarantine, staying at home, wearing masks, and physical distancing. These large-scale life changes might alter society’s psychological burden.

The COVID-19 pandemic has led to a high prevalence of psychological problems [3, 4] and the public has exhibited anxiety-related behaviors based on social rejection as well as the stigmatization of people affected by the virus [5, 6]. Psychological distress (PD) is a condition with a specific set of symptoms that consists of all non-specific changes in a living system [7], including multifactorial unpleasant emotional experiences of a psychological (i.e., cognitive, behavioral, emotional), social, and/or spiritual nature [8]. Being an important comorbidity for PD, anxiety might affect health outcomes [9]. Although other potential risk factors for PD during the COVID-19 pandemic have been identified, including economic hardship, presence of comorbidities, and quarantine [10, 11], we do not yet fully understand the background dynamics of human society related to the pandemic.

In Japan, the first nationwide governmental declaration of emergency was instituted from April 16, 2020 to May 25, 2020 in response to the spread of COVID-19. As of May 31, 2021, the number of confirmed cases of COVID-19 was 744,487, and 12,967 deaths were attributed to the disease [12]. The infection control behaviors of people and communities in Japan depended on public self-restraint in compliance with governmental requests [13]. In fact, most people complied with COVID-19 measures by engaging in physical distancing, mask-wearing, and handwashing [14]. Meanwhile, there were inappropriate behaviors such as people racing to buy surgical masks at pharmacies, which caused mask shortages in hospitals, and misinformation about the pandemic, which led to prejudice against patients and their families [15].

Previous studies have suggested that local social dynamics might be a powerful predictor of PD. For instance, social cohesion is associated with decreased PD [16, 17] and social stressors may negatively affect psychological health [18, 19]. During the COVID-19 pandemic, some observational studies have found factors associated with psychological problems, including anxiety and fear of COVID-19, as well as PD [20–23]; however, the relationship between these factors and any changes in the COVID-19 pandemic have not been fully elucidated. To understand the extent of any changes in the community, and people’s lives, behaviors, and perceptions, a qualitative study design is useful for identifying unknown sources within the broader social context [24]. In addition, by implementing a mixed-method approach that integrates quantitative and qualitative data, a comprehensive understanding can be realized that takes into consideration the aims of the research [25].

In this context, the present study seeks to identify specific life changes that correlate with PD during the COVID-19 pandemic. We assume that there might be unknown factors involved in the association between PD and life changes at the individual level. We furthermore assume that such factors might discernible if the direct effect of the COVID-19 pandemic and socioeconomic factors could be excluded. Therefore, this study involved company employees and civil servants whose workplaces had no confirmed cases of COVID-19 and whose neighborhoods had only a few cases. Moreover, a mixed-method approach was adopted to examine the association of PD with quantitative related factors and qualitatively described life changes in a population of employed Japanese.

## Materials and methods

### Study design and participants

**Fig 1:**
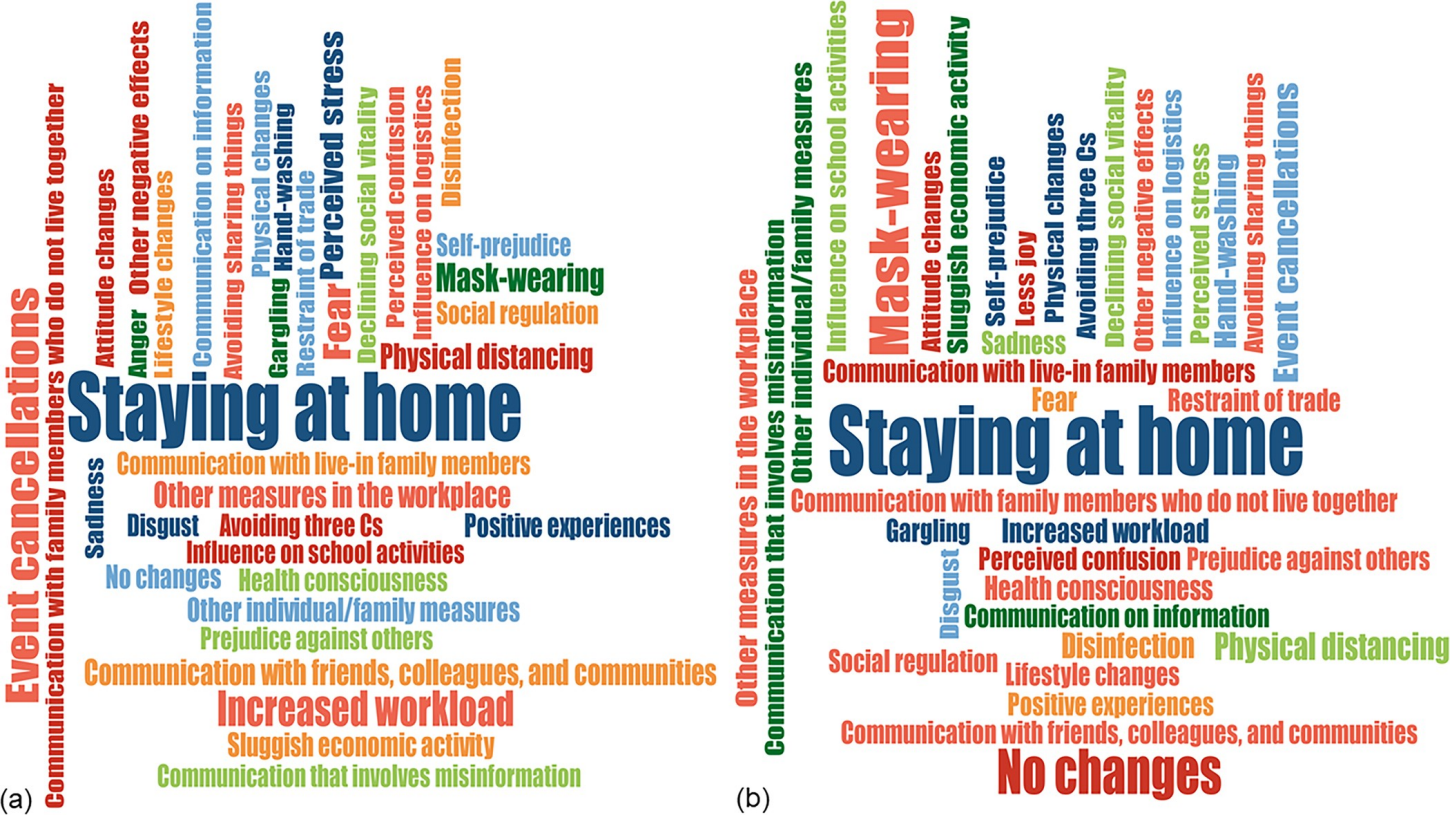
Flowchart of participant enrollment in the study.

This cross-sectional study applied a convergent mixed-method approach to investigate the relationship between specific life changes and PD during the COVID-19 pandemic in comparison with individuals without PD. We recruited participants from local institutions, including a company (corporate workers) and a city government (city officials), in Niigata, Japan. Corporate workers who participated in this study were regular employees of an information and telecommunication facility construction company affiliated with the Niigata office, whereas city officials included city hall staff and staff of the city hospital, city fire departments, and city nursery. This hand-distributed baseline survey was conducted in July 2020 and completed by 623 participants (response rate, 42.8%). As of July 31, 2020, Niigata Prefecture had 111 COVID-19 patients (80 in Niigata City), whereas the city to which the city officials belonged had no confirmed cases. A flowchart showing participant enrollment in the study is shown in Fig 1. After excluding participants with missing data, 609 participants were analyzed quantitatively, and of these, 438 were included in qualitative and convergent analysis. All procedures were approved by the Ethics Committee of Niigata University (Approval No. 2020–0030).

### Questionnaire description and coding procedure

A questionnaire was used to assess demographic characteristics, job operations (only usual operations, mainly COVID-19-related operations, or both), and individual preventive measures for COVID-19 transmission. PD was assessed by the Japanese version of the Kessler Psychological Distress Scale (K6) [26, 27], which consisted of six simple items measuring how frequently a participant felt sad, nervous, restless, hopeless, and/or worthless during the previous 30 days. Each item is rated on a 5-point scale, from 0 (*none of the time*) to 4 (*all of the time*). Total scores were the sum the six item scores and thus ranged from 0 to 24, with a score of 13 or above defined in this study to indicate PD [28]. The questionnaire included the following open-ended question: “What has changed before and after the COVID-19 pandemic for each of the following: yourself, your family, your work, and your community? Please freely describe what you feel has changed.”

The coding procedure began with systematic open coding for subsequent categorization of trends and patterns of words, structures, and abstraction. The coding process was performed by two authors (the primary researcher and a research assistant). The first step was conducted independently across the authors. After discussion of differences in coding agreement, code definition, and categorization, a second round was conducted, after which inter-coder agreement was assessed (coefficient kappa = 0.64). Table 1 shows the final coding descriptions. We allowed multiple coding for the same response because single respondents often provided multiple answers to one question (e.g., responses to life changes in oneself [“I started to wear a mask, disinfect things, and ventilate the room”] have three codes for infection control measures).

**Table 1:** Categories, codes, descriptions, and examples of life changes due to the COVID-19 pandemic.

Categories	Codes	Descriptions	Examples of responses
Infection control measures	Mask-wearing/hand-washing/disinfection/gargling	Individual anti-infection measures	“Wear a mask outside and wash my hands and gargle when I get home”
	Other individual/family measures; Other measures in the workplace	Non-specified anti-infection measures taken by individuals/families or at work	“Measures taken in the workplace,” “Family members are taking measures to prevent the spread of infection”
	Staying at home	Avoiding outings, travelling, and eating out	“No more unnecessary outings”
	Avoiding the three Cs	Avoiding closed spaces, crowded places, close-contact settings	“Avoid three Cs,” “Avoid crowded places and busy times”
	Physical distancing	Physical distancing, including personal interactions	“Avoid contact with people as much as possible,” “Schedule online meetings”
	Event cancellations	Event cancellations, including gatherings and community meetings	“Cancellation or reduction of events,” “Cancellation of festivals”
	Social regulation	Governmental social measures (other than event cancellations), including measures in public places or stores.	“Following city government measures to stop COVID-19,” “Cease all community activities”
Health care compliance	Attitude changes	Changes in attitudes or feelings towards family, work, and the community.	“I no longer feel uncomfortable with infection prevention,” “I feel the difference between those who are aware of infection control and those who are not”
	Lifestyle changes	Changes in daily life, infection control becoming a daily practice, transition to a new normal lifestyle.	“My lifestyle has changed,” "I’m leading a new kind of life,” “Remote classes have disrupted my kids’ daily routine”
	Health consciousness	Change in awareness of one’s overall health condition.	“I’ve become more health conscious,” "I’m taking my temperature every day,” “I try to have to a balanced diet”
	Physical changes	Changes that happened to their body.	“I gained weight from lack of exercise,” “My skin has become irritated from wearing masks all the time.”
Positive experiences	Positive experiences	Experience positive feelings, joy, and positive situation	“Enjoy dressing up in style with a handmade-mask”
No changes	No changes	Feeling no changes due to COVID-19	“No changes in my life,” “Nothing”
Communication	Communication with friends, colleagues, and communities	Changes in methods of communication and communication status with friends, family members, etc.	“I feel like there is less communication with friends, colleagues, and community members since the pandemic”
	Communication with live-in family members	Changes in methods of communication and status with live-in family members	“More time spent together at home,” “More time spent with live-in family”
	Communication with family members who do not live together	Changes in methods of communication and status with non-live-in family members	“Not being able to meet with children who do not live at home or distant relatives”
	Communication that involves misinformation	Describing fake news and rumors	“I feel like there is more fake news and rumors,” “Rumors of COVID-19 cases in the community were confirmed.”
	Communication on information	Communication on information about COVID-19	“Discuss COVID-19-related information,” “Collect COVID-19-related information”
Financial influences	Increased workload	Increased workload due to COVID-19 response.	“Increased COVID-19-related workload,” “Spend a lot of time on infection prevention measures at work”
	Restraint of trade	Compliance with government-requested self-restraint	“Sports facilities are closed due to self-restraint request,” “Shorter restaurant hours”
	Influence on logistics	Shortage of anti-infection products in communities	“Lack of masks and disinfectant,” “Increased price of masks”
	Sluggish economic activity	Suggest sluggish personal and family activities and institutional activity	“Less work and sales,” “Stores are closed due to COVID-19”
	Influence on school activities	Items related to school closure and other influences on student learning, school events, and extracurricular activities	“School activities are cancelled or restricted,” “Less learning due to school closures”
Perceived prejudice	Prejudice against others	Prejudice against others and people outside the community	“I think people who don’t wear masks are dangerous,” “I don’t like cars with out-of-prefecture plates”
	Self-prejudice	Feeling prejudice based on your behaviors and engaging in compulsive behaviors.	“Difficult to see distant friends and family,” “Without a mask, people look at me funny”
Emotion and perceptions	Perceived stress	Perceived stress	“Feeling stressed because I cannot go outside,” “Feeling stressed because I cannot do what I love to do”
	Perceived confusion	Perceived confusion about workplace and social measures regarding COVID-19	“I can’t help myself and I can’t do anything about it,” “Having trouble getting out of the province”
	Declining social vitality	Visible social vitality	“Vibrancy of the community has been lost,” “No one is outside”
	Less joy/fear/sadness/disgust/anger	Fear includes anxiety, concern, and nervousness. Disgust includes pain and exhaustion	“Fear of getting infected,” “Frustrated I cannot go out,” “Fun is gone”
	Other negative effects	Feeling difficulties, inconvenience, fatigue and other negative effects related to infection prevention	“No rest and little sleep,” “Too hot to wear a mask,” “Inconvenient to go shopping”

### Analyses

Quantitative hypothetical testing analyses were calculated by SAS statistical software (version 9.4, SAS Institute Inc., Cary, NC). Qualitative and mixed-method analyses were performed using MAXQDA 2020 Analytics Pro (VERBI Software GmbH, Berlin, Germany).

Participants’ background characteristics were presented as numbers (percentages). The difference concerning categorical background characteristics and codes between participants with PD and those without were assessed by the chi-square test. The analysis took a qualitative content and thematic analysis approach [29, 30]. Content analysis was performed collating codes according to participants’ perceived life changes due to the COVID-19 pandemic with respect to themselves and their family, job, and community. Spearman’s rank correlation coefficients were calculated between each code. For thematic analysis, we sought major themes from a population potentially vulnerable to PD in order to identify the in-depth context of the impact of pandemic-related life changes on PD.

## Results

Table 2 shows the participants’ descriptive characteristics. In total, 609 participants (66.9% male) completed the questionnaire, including the K6 score. Mean age and median K6 score was 45.3 years old (SD 12.7) and 3 (interquartile interval [IQI] 0, 8), respectively. There were no confirmed cases of COVID-19 in the study population. Participants with PD (54; 8.9%) were more likely to be women (*P* = 0.016), and younger (*P* < 0.001) than those without PD.

**Table 2:** Descriptive characteristics according to Kessler-6 score.

	Overall (n = 609)	Kessler-6 score		*P*-value
		<13 (n = 555)	13 or more (n = 54)	
Age, years				<0.001
< 30	81 (13.8)	69 (12.9)	12 (22.6)	
30–39	118 (20.1)	108 (20.2)	10 (18.9)	
40–49	156 (26.5)	133 (24.9)	23 (43.4)	
50–59	138 (23.5)	131 (24.5)	7 (13.2)	
≥ 60	95 (16.2)	94 (17.6)	1 (1.9)	
Male sex	392 (66.9)	365 (68.4)	27 (51.9)	0.016
Affiliation				0.167
Company employee	280 (46)	260 (46.9)	20 (37)	
Civil servant	329 (54)	295 (53.2)	34 (63)	
Education				0.307
Junior high or high school	220 (36.3)	204 (36.9)	16 (30.2)	
Junior college	191 (31.5)	176 (31.8)	15 (28.3)	
University or higher	195 (32.2)	173 (31.3)	22 (41.5)	
Job operations				0.992
Only usual operations	304 (50.2)	278 (50.3)	26 (49.1)	
Mainly COVID-19-related operations	19 (3.1)	17 (3.1)	2 (3.8)	
Both	259 (42.7)	236 (42.7)	23 (43.4)	
Others	24 (4)	22 (4)	2 (3.8)	

*Note*. Data are shown as numbers (percentages). *P*-values are calculated by chi-squared test. Number of participants to analyze decreased by 21 for age, 23 for sex, 3 for education, and 3 for job operations.

### Content analysis comparing respondents with psychological distress and those without psychological distress

Of the 609 participants, 438 answered the open-ended question about pandemic-related life changes for themselves and their family, workplace, and community. There were 389 responses about themselves, 359 about their family, 367 about their workplace, and 318 about their community. Spearman’s correlation coefficients between each code is shown in S1 Table. Infection control measures, including “mask-wearing,” “handwashing,” “disinfection,” and “gargling” were correlated with each other (Spearman’s correlation coefficients 0.243–0.575) (S1 Table). The correlation coefficients between “other individual/family measures” and “other measures in the workplace,” “physical change” and “restraint of trade,” and “communication with friends, colleagues, and communities” and “communication with live-in family members” were 0.318, 0.310, and 0.263, respectively. That between “communication that involves misinformation” and “sadness” or “anger” was 0.252 or 0.283, respectively.

**Fig 2:**
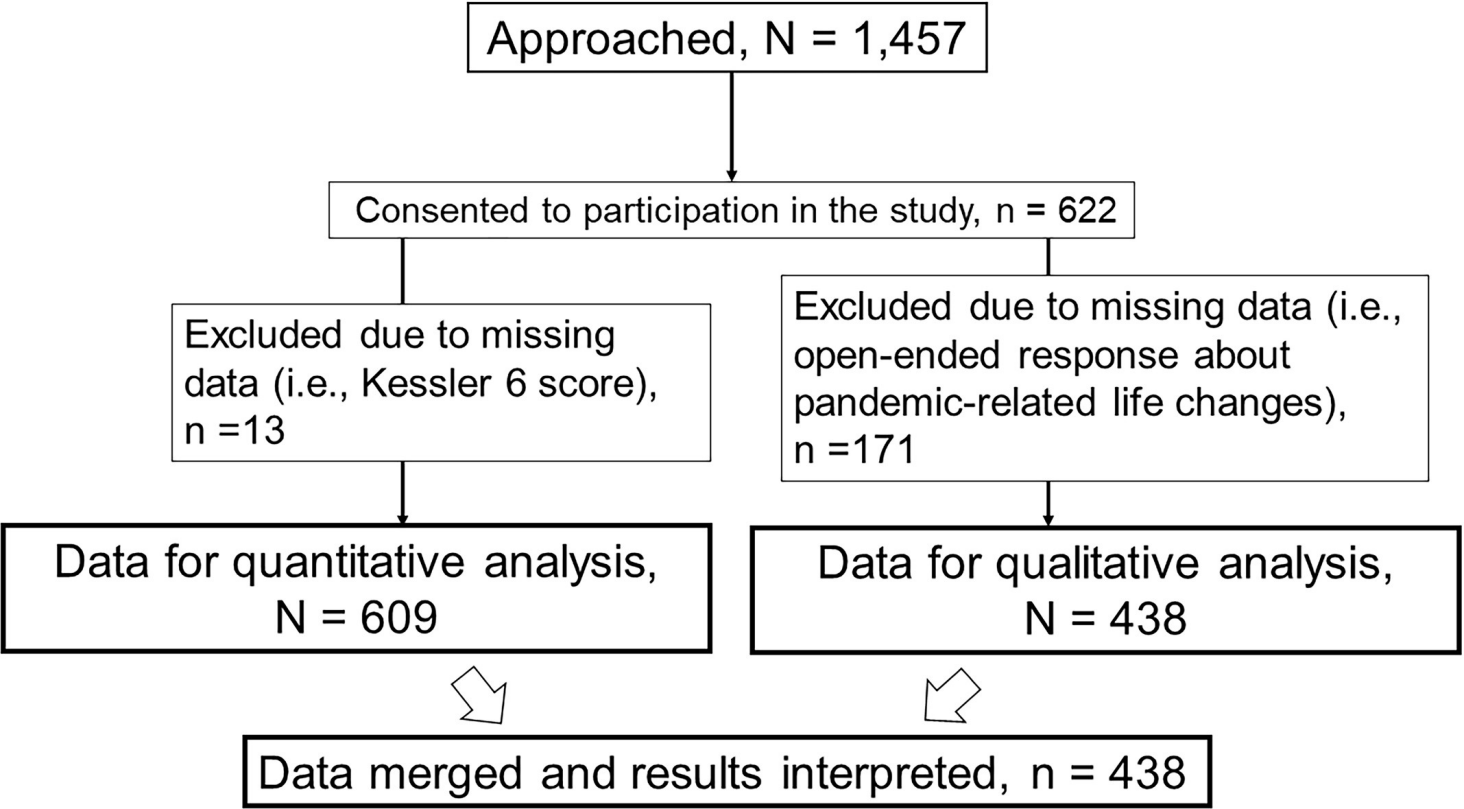
Code clouds of pandemic-related changes. Participants with psychological distress (a) and those without (B).

Among respondents to the open-ended question about life changes, PD was observed in 36 (8.2%). Figs 2 and S1 show frequency code comparisons in respondents with PD and those without. Fig 2 shows that “staying at home” was the most frequent code in respondents with and without PD whereas the subsequent frequent codes were differently distributed between those with and without PD. In this content analysis, “event cancellations” and “increased workload” were the next most frequent codes for respondents with PD compared with “mask-wearing” and “no changes” for respondents without PD (S1 Fig). “Increased workload” (*P* < 0.001), “mask-wearing” (*P* = 0.014), “no changes” (*P* = 0.001), “event cancellations” (*P* = 0.035), “communication with friends, colleagues, and the community” (*P* = 0.039), and “positive experiences” (*P* = 0.048) were significantly different between respondents with PD and those without. Subsequently, we performed a similar content analysis regarding emotion and perception categories among respondents with PD and those without. The top three most frequent codes were “fear,” “perceived stress,” and “other negative effects” for respondents with PD and “fear,” “other negative effects,” and “declining social vitality” for those without PD (S1 Fig). Of these codes, “perceived stress” (*P* < 0.001), “anger” (*P* < 0.001), and “fear” (*P* < 0.001) were significantly different between respondents with PD and those without.

### Thematic analysis of the subgroup with more frequent psychological distress

To seek an in-depth understanding of the association between life changes and PD, we performed a thematic analysis among women and younger respondents (under 40 years old) because they were more likely to have PD in this study (Table 2). As a potentially vulnerable population in terms of PD, identified themes might help elucidate the major contexts underlying the distress they felt.

#### “Frustration with increased workload and struggles with stress relief”

Distressed women (*n* = 19) were more likely to consider that major life changes had increased their workload (16.7%) and to feel fear (18.2%) compared with distressed men (7.1% and 3.6%, respectively). Among the distressed younger cohort (*n* = 13), the top three life changes were “staying at home” (23.3%), “event cancellations” (16.3%), “increased workload” (9.3%), “sluggish economic activity” (9.3%), and “fear” (9.3%). Several women and the younger cohort mentioned that despite frustration over increased workloads related to workplace infection control measures, they also could not go out without concern, which served to increase their stress.

#### “Concern about relatives”

This theme was especially evident in women (e.g., “*I’m worried about my children and parents who live away from home*.*”)*. Women tended to express more concern about other people, such as family members and particularly children who lived away from home. In fact, several women mentioned concerns about the effects of school closures on their children’s lives during the state of emergency. Also, because other members of the family had refrained from going out, these women seemed to be concerned about their sedentary lifestyles.

#### “Desire to achieve work satisfaction when dealing with infection control”

Distressed younger respondents frequently mentioned less work satisfaction due to the excessive burden of COVID-19-related measures. Infection control measures were perceived to interfere with their sense of work-related satisfaction. Several respondents mentioned that infection control measures prevented them from providing satisfactory service to customers, causing them to feel “*[the] need to develop new ways to achieve work satisfaction when dealing with infection control*.” Furthermore, respondents mentioned a tense workplace atmosphere and changed workplace layout due to physical distancing, which suggests less communication.

## Discussion

This convergent mixed-method study found that “staying at home” was considered the biggest life change due to the COVID-19 pandemic among all respondents (with and without PD). Pandemic-related life changes that were significantly different between respondents with PD and those without were mainly “increased workload” and “no changes.” In terms of emotional and perceptual changes, “perceived stress,” “anger,” and “fear” were related to PD. Moreover, the results of this study identified major themes among a potentially vulnerable population (women and younger people) that might allow us to understand unknown processes behind the determinants of specific life changes related to PD during the pandemic.

Most participants complied with preventive measures for COVID-19 transmission (i.e., staying at home, mask-wearing, handwashing), even in the absence of laws requiring such compliance and few COVID-19 cases in their neighborhoods. Given that this result is consistent with other Japanese studies [14, 23], pandemic-related behavioral changes might represent the collectivistic nature of the nation of Japan. Collectivistic cultures are interdependent, and so most people take precautions against infectious diseases through self-restraint, even in the absence of laws as has been the case in Japan. Given that engaged emotions and relational harmony can be a predictor of well-being in an interdependent society [31, 32], “staying at home” may not correlate with PD.

Notably, “increased workload” and “no changes” were differently distributed between respondents with and without PD. Given that the study population included workers, “increased workload” might be expected because the government recommended the implementation of precautionary measures at each workplace. However, those measures proved to be a huge burden not only at the workplace but also in daily life [33, 34]. A previous study in Japan found an inverse association between the number of workplace measures (0–23) and employees’ PD as assessed by the Brief Job Stress Questionnaire [35]. Indeed, in the present study, some respondents mentioned that they had been assigned to carry out COVID-19 control measures as part of their regular work, thereby increasing their workload. Moreover, a strained workplace atmosphere was described as a life change, as shown in a previous study that found an effect of job stress on PD [36]. Even with few confirmed COVID-19 cases in the community, unbalanced control measures and additional COVID-19-related tasks might lead to increased workloads rather than decreased anxiety. In contrast, respondents who did not perceive any pandemic-related life changes were unlikely to feel distress. These respondents might have psychological flexibility, which is a process of acceptance and commitment therapy that consists of defusion, acceptance, present moment awareness, self-as-context committed action, and values [37]. Psychological flexibility is a widely recognized and important factor for well-being across conditions and populations [38, 39], and therefore could buffer PD during a pandemic.

Historically, infectious diseases have been the greatest cause of human death. Thus, a pandemic could be a threat to people, leading to negative emotions such as fear, anger, and stress. In fact, several studies have found that negative emotion is a predictor of PD [40–43]. Some respondents mentioned fear of uncertainty for the future but also fear of bothering others or fear of how others might think of them if they or their family members were to become infected and/or transmit the virus themselves. As a similar life-change context, a collectivistic society might impact this relationship because social identity can help manage threats [44] and group commitment [45]. However, collective behavior can magnify people’s fears when sociocultural or traditional approaches to disease control collapse. For instance, normative values might negatively affect psychological health if they are perceived as obligations [46].

Consistent with other studies on associated factors of PD [47–49], the present study identified women and younger people as a vulnerable population for PD. Among them, we identified the following major themes related to PD during a pandemic: *(1) Frustration with increased workload and struggles with stress relief*; (2) *concern about relatives; and (3) desire to achieve work satisfaction when dealing with infection control*. A collective universal behavioral campaign might cause excessive workloads in a collectivistic culture where people cannot violate in-group commitments regardless of stress. Greif, et al, found that overly strict measures such as lockdowns increased stress [18]. Although social connection during a pandemic can help increase uptake ofbehavioral changes that can be either harmful or beneficial, it can also help people manage their emotions and remain resilient during difficult times [50, 51].

Moreover, PD might be caused by female workers’ anxiety or concerns about those they give care to. A large body of literature has examined caregivers’ psychological health [52–54]. Also, an imbalance in work and home may cause PD among workers [55]. However, the construct of work-family balance such as work-family conflict could mediate PD as well as correlate with work and life satisfaction [56, 57]. To maintain psychological well-being beyond the COVID-19 pandemic, it is important to feel life (work) satisfaction when dealing with disease (infection) control. Coping well with stress and elevating motivation for life is crucial to reducing psychological burdens during a pandemic. Taken together with our results, these findings suggest that it might be beneficial to combine the implementation of infection control measures with appropriate distress-relieving techniques.

To our knowledge, this is the first study to identify specific life changes due to the COVID-19 pandemic that are associated with PD, regardless of the number of cases in the community. The strengths of this study include having regularly employed workers, including city officials, as a study population, to eliminate the influence of pandemic-related financial changes on PD given the association between financial difficulties and PD [23, 58]. Therefore, this study enabled us to understand social and individual contexts without economic factors that might bias the relationship with PD. Also, the study population was recruited from a local community that had few or no confirmed COVID-19 cases as of the study period (July 2020), which permitted examination of general psychological responses apart from the direct effect of the virus.

Although these strengths highlight the robustness of the results, several limitations should be noted. First, a mixed-method approach has been established and allows for better understanding of real-life contexts, although this strategy is still under discussion [59]. We analyzed integrated data from a constructive perspective, but interpretation of the result requires caution due to difficulties ensuring complete elimination of subjectivism. Second, the present study was limited by a homogenous sample from a local community in Japan. Our results might cover cultural aspects of Asian countries, but not capture the full picture of PD during the COVID-19 pandemic worldwide. Also, we should acknowledge the limitation that we did not use a COVID-19-specific questionnaire, such as the Fear of COVID-19 Scale [60, 61]. Third, qualitative data was solely based on text-based responses to an open-ended question and not on interview or focus group procedures, which would have provided more in-depth information on life changes. Fourth, the study did not conduct satisfactory quantitative hypothesis testing such as multivariable-adjusted analyses, which could confound the results. Finally, causality and temporality cannot be determined in a cross-sectional study.

## Conclusion

This mixed-methods study provided a more nuanced picture of the relationship between people’s life changes and PD during the COVID-19 pandemic in community-dwelling Japanese workers. Overall, participants considered “staying at home” their biggest life change, suggesting that they implemented this behavioral change through collective social commitment. The present study identified specific life changes and vulnerable characteristics related to PD. Notably, identified themes in this study seem to provide a source for mediating PD in a community during a pandemic, such as the importance of social acceptability when balancing infection control measures with relief of negative emotions. This evidence might help mitigate PD due to the COVID-19 pandemic as well as in future public health crises involving infectious diseases.

## Supporting information

S1 Table Spearman’s rank correlation coefficients between each code. (XLSX)

S1 Fig Code distribution of participants with psychological distress and those without. *Note*. *Indicates significant difference between participants with psychological distress and those without (*P* < 0.05).
(TIF)
