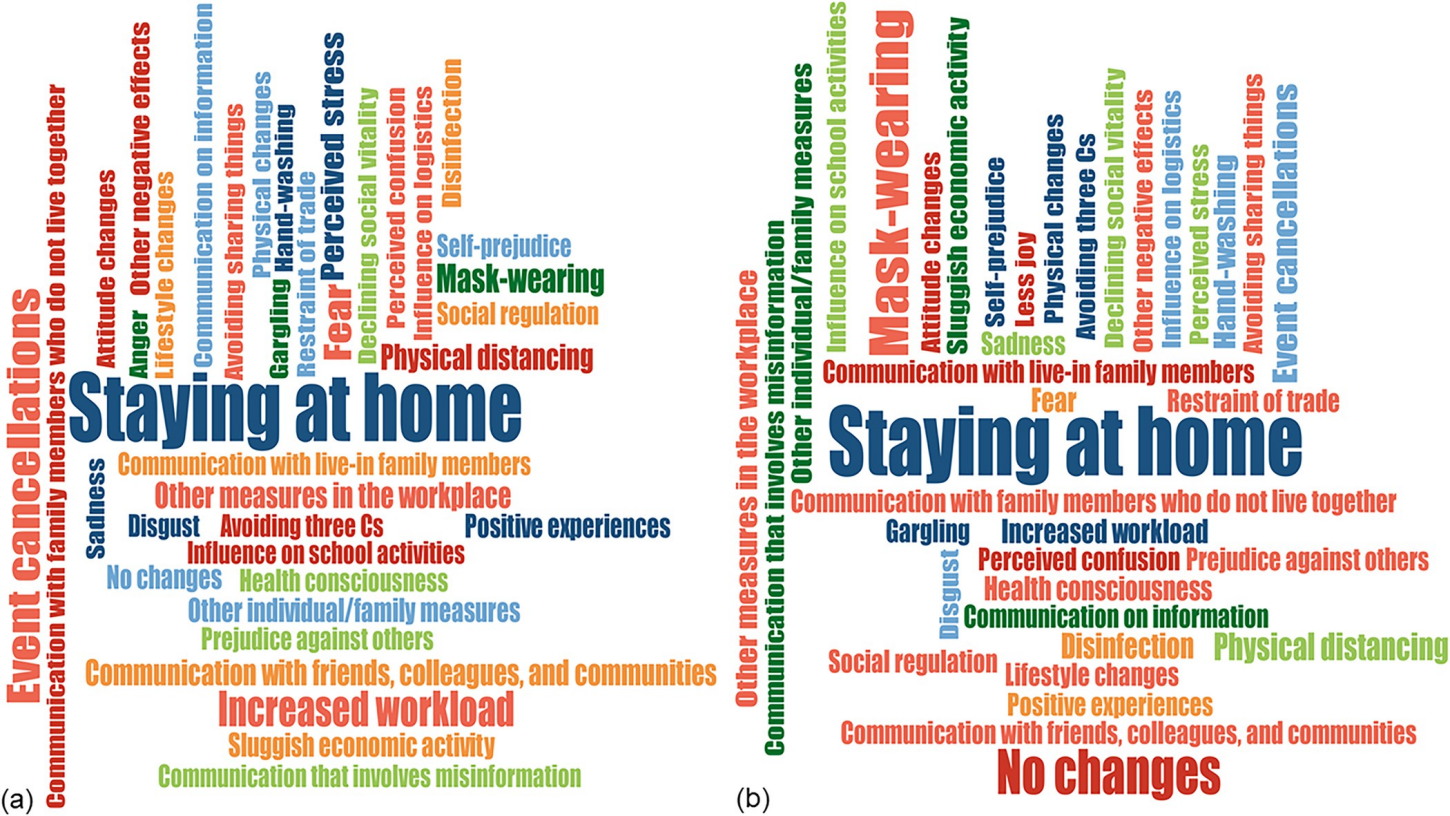# Correction: Determination of specific life changes on psychological distress during the COVID-19 pandemic

**DOI:** 10.1371/journal.pone.0308278

**Published:** 2024-07-31

**Authors:** Keiko Kabasawa, Junta Tanaka, Tomoyo Komata, Katsuhiro Matsui, Kazutoshi Nakamura, Yumi Ito, Ichiei Narita

The images for Figs 1 and 2 are incorrectly switched. The image that appears as Fig 1 should be Fig 2, and the image that appears as Fig 2 should be Fig 1. The figure captions appear in the correct order.

**Fig 1:**
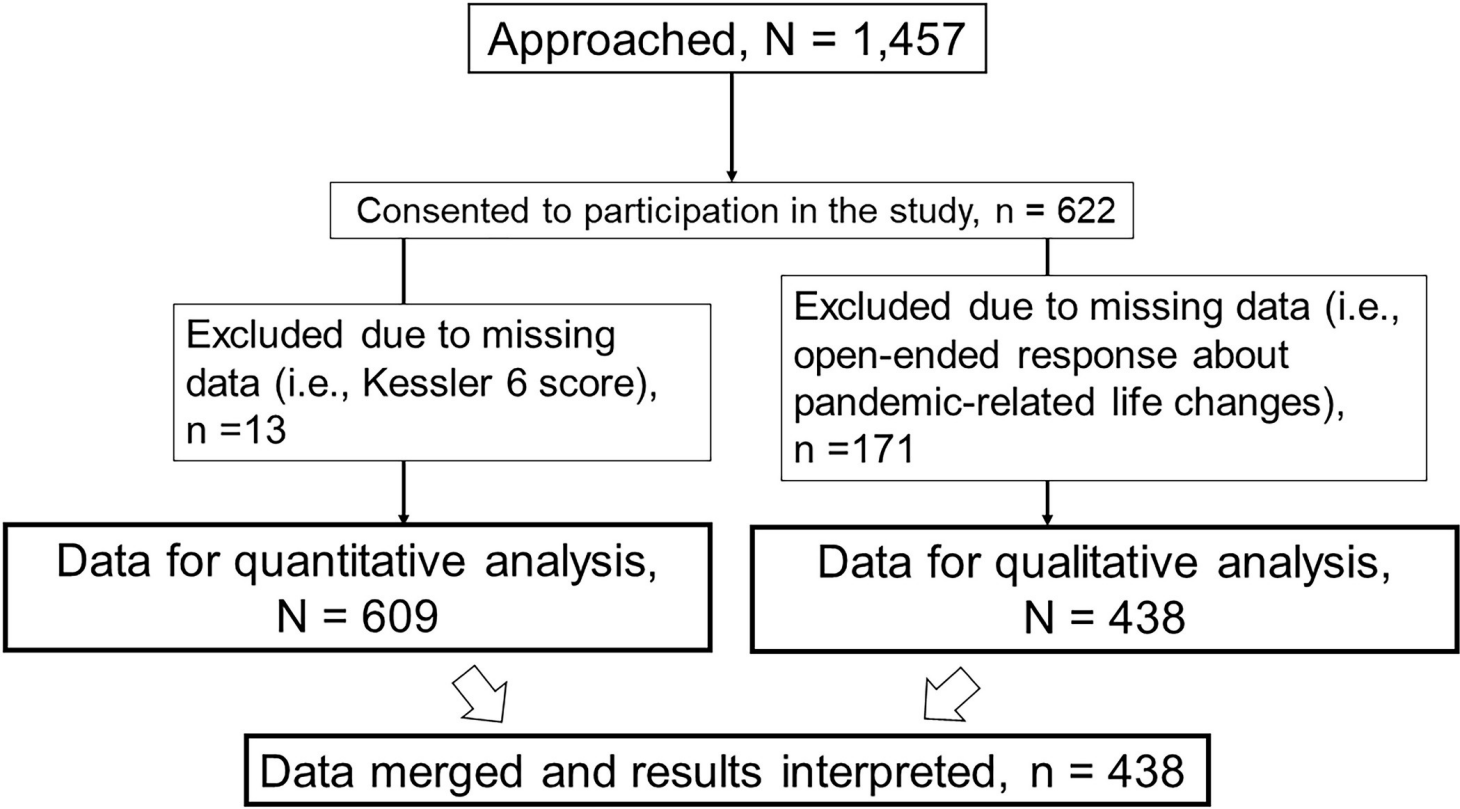


**Fig 2:**